# Challenging Medical Ghostwriting in US Courts

**DOI:** 10.1371/journal.pmed.1001163

**Published:** 2012-01-24

**Authors:** Xavier Bosch, Bijan Esfandiari, Leemon McHenry

**Affiliations:** 1 Department of Internal Medicine at the Hospital Clínic and the Institut d'Investigacions Biomèdiques August Pí i Sunyer, University of Barcelona, Barcelona, Spain; 2 Law Firm of Baum, Hedlund, Aristei & Goldman, Los Angeles, California, United States of America; 3 Department of Philosophy, California State University, Northridge, California, United States of America

## Abstract

Xavier Bosch and colleagues expand upon a recent analysis by Simon Stern and Trudo Lemmens in *PLoS Medicine* and outline areas in which authors participating in medical ghostwriting could be held legally liable.

**Summary Points**

- Despite growing concern about medical ghostwriting, pharmaceutical companies, universities, medical journals, and communication companies employing ghostwriters have thus far failed to adequately stem the problem. As a result, some commentators have proposed that legal remedies could be sought by patients harmed by drugs publicized in ghostwritten papers.
- In this Essay, we build on a recent analysis by Stern and Lemmens in *PLoS Medicine* to outline specific areas of legal liability.
- For example, when an injured patient's physician directly or indirectly relies upon a journal article containing false or manipulated safety and efficacy data, the authors, including guest authors, can be held legally liable for patient injuries.
- In addition, guest authors of ghostwritten articles published by Medicare- and Medicaid-recognized peer-reviewed medical journals used as clinical evidence for indications for off-label uses may be liable under the federal False Claims Act for inducing the United States government to reimburse prescriptions under false pretenses.
- Paying guest authors of ghostwritten papers may influence clinical judgment, increase product sales and government health care costs, and put patients at risk by misrepresenting risk-benefit. Therefore, both physicians and sponsor companies may be liable under the federal Anti-Kickback Statute.
- Although guest authors and pharmaceutical defendants may argue a First Amendment right to participate in ghostwriting, the US Supreme Court has firmly held that the First Amendment does not shield fraud.

## Introduction

Complaints about the ethics of medical ghostwriting have increased in the last decade, but little has changed [1]–[14]. Corruption of the scientific literature through ghostwriting persists in medicine due to the enormous profits for all stakeholders [15], including the pharmaceutical industry that creates the publication strategy, academic researchers acting as key opinion leaders (KOLs) for industry, universities employing KOLs, medical journals and their proprietors, including medical societies and publishers, and medical communication companies employing ghostwriters.

Ghostwriting openly infringes academic standards and, in many cases, as recently argued by Stern and Lemmens in *PLoS Medicine*, contributes to fraud [16]. Typically, the practice involves industry-financed writers generating articles that either promote the sponsor company's products or discredit competing ones, with eventual authorship credited to academic researchers who provide little or no input, thereby concealing industry involvement and contributing to distorted drug profiles. In the United States, cases relating to gabapentin [17], rofecoxib [2], paroxetine [7], sertraline [18], fenfluramine/phentermine (fen-phen) [19], and Prempro [3] are well documented, while many others, relating to rosiglitazone, olanzapine, quetiapine, valdecoxib, and celecoxib, remain under seal by the courts. These cases demonstrate the dangers inherent in permitting pharmaceutical companies to maintain the status quo.

Some editors, fully aware that ghostwritten manuscripts are submitted to their journals, refuse to police their content [20]. Although other journals, most notably *PLoS Medicine*, as well as several editors' associations have produced policies against the practice, in some cases adopting clear and visible positions, little has changed [1],[6],[11],[21]–[23]. In addition, despite efforts to reinforce authorship and publication requirements, journals' responses to ghostwriting remain unsatisfactory, as shown by a recent study of 630 articles from six high impact medical journals [24]. In 2008, the overall prevalence of articles with honorary authorship, ghost authorship, or both, was 21.0%, which represented a decline from 29.1% in 1996. Although the prevalence of ghost authorship showed a significant decline, there was no change in the prevalence of honorary authors relative to 1996 [24]. This study concluded that inappropriate authorship remains a significant problem in high impact biomedical publications.

Indeed, even the policies adopted by the International Committee of Medical Journal Editors have failed to clarify how the corruption of medical literature could be curtailed [14]. Substantial contribution to manuscript design or drafting is of little significance when marketing messages are planted in the ghostwriter's first draft well before a nominal author is selected. Authors may give approval when the paper is submitted for publication, but this only occurs after the sponsor company has ensured the manuscript meets its marketing goals and the legal department has transferred ownership to the submitting author. The manuscript and message are therefore controlled by the company rather than the nominal authors [3],[7].

Since self-regulation has not produced results and the government has failed to have any significant impact, we argue that the only remaining option is the legal system. Building upon the recent Stern and Lemmens article that proposed viewing ghostwriting as fraud [16], this Essay expands on the possible legal remedies for medical ghostwriting that can help outlaw a practice that has long tainted journal content and jeopardized patient safety.

## Legal Remedies for Medical Ghostwriting

Stern and Lemmens recently advanced various legal theories under which “guest authors” can be held accountable, including filing an action under the Racketeer Influenced and Corrupt Organizations Act (RICO) [16]. They opined that monetary damages could include a reduction in the subscription value of the journal publishing ghostwritten articles. They concede individual damages would be nominal but suggest that potential liability and reputational harm may curb ghostwriting. We endorse this novel theory and the other theories (i.e., fraud on the court) Stern and Lemmens advance. We question, however, whether a law firm would undertake the Herculean task of filing RICO actions against guest authors when the potential of recovery (even if aggregated and trebled) would be, in litigation values, fairly modest and nominal. In reality, the transactional costs of such an endeavor, the novelty of the theories, and the nominal damages at issue would likely discourage law firms from prosecuting such cases.

We fully agree with Stern and Lemmens that the legal system could be effective in curbing ghostwriting, and suggest the following models of liability.

## Personal Injury and Wrongful Death Damages Caused by the Guest-Authors' Misrepresentations

Guest authors lending their names to ghostwritten articles touting the safety and efficacy of a drug have an independent duty to exercise ordinary care and prevent injury to others as a result of their conduct [25]. As the influential Justice Benjamin Cardozo noted long ago: “It is ancient learning that one who assumes to act, even though gratuitously, may thereby become subject to the duty of acting carefully, if he acts at all.” [26] When US courts have considered misrepresentations that implicate a risk of physical harm to others, they have often looked to the rules set forth in the Restatement Second of Torts, sections 310 and 311 [27].

Sections 310 and 311 of the Restatement allow injured third parties to recover from a person who has made an intentional and negligent misrepresentation inducing action that involves a risk of physical harm [28]. The Restatement emphasizes that liability “extends to any person who, in the course of an activity which is in furtherance of his own interests, undertakes to give information to another, and knows or should realize that the safety of the person or others may depend on the accuracy of the information” [29].

The following hypothetical case, which is applicable to many real world events, illustrates how liability can be established: A drug manufacturer conducts a study whose primary endpoints show the study drug poses serious risks and is not effective. The drug manufacturer manipulates the data and creates post hoc secondary and tertiary endpoints to create favorable outcomes. Once a favorable outcome is created, the manufacturer hires a ghostwriting firm to draft an article falsely touting the purported benefits of the drug and failing to disclose the side effects. The manufacturer then retains various KOLs and reputable university professors to lend their names and credentials to the drafted article. The article is published in a widely read medical journal and physicians begin to prescribe the drug in reliance on the article's claims and the “authors'” reputations. Placebo responses help mask efficacy issues, but some patients begin to suffer from the undisclosed side effects—which in many cases cause serious and fatal injuries. Under these circumstances, the injured patients and their families sue the manufacturer for injuries and death caused by the drug's side effects. The guest authors, however, are never named as defendants. We argue that when an injured patient's physician directly or indirectly relied upon a journal article containing false/manipulated safety and efficacy data, then pursuant to the legal authority outlined above, the authors of that article, including guest authors, are legally liable for patient injuries and could be named as defendants.

Accordingly, guest authors should know that the information to which they have affixed their name (and purportedly “authored”) will be relied on by other medical professionals to make treatment decisions and that, should the information be false, the patients receiving the drug are placed in peril. Guest authors cannot claim immunity from the law by stating that they relied on data summaries presented by the pharmaceutical company. Such facts would fall squarely within the elements of liability outlined in Sections 310 and 311 of the Restatements discussed above. Even if the prescribing physician never actually read the ghostwritten article but its messages were relayed to him or her by colleagues, under established case law, the prescriber can be deemed to have relied on the ghostwritten article [30]. We therefore recommend that in cases where patients are harmed as a result of a pharmaceutical manufacturer's fraudulent representations involving ghostwritten articles, serious consideration should be given to naming as defendants the guest authors who lent their names to the misrepresentations.

In a few current pending pharmaceutical mass-tort litigations, plaintiffs' lawyers have begun naming ghostwriting firms such as Excerpta Medica, Inc. and Elsevier, Inc. as defendants [31], although we are aware of no published decisions or cases in which guest authors have been named as defendants. The transactional costs involved in naming guest authors as defendants are minimal given that a suit will already be pending against the manufacturer. The potential damages at issue can be significant and will depend on the plaintiff's injury and the egregious nature of defendants' conduct. Such potential liability serves four purposes. First, guest authors will be held accountable for their fraud and negligent conduct. Second, they will be confronted with the consequences of their actions and will have to answer at pre-trial depositions and at trial. Third, this will, we hope, force guest authors to review the data and independently confirm the conclusions prior to lending their names to articles drafted for them by manufacturers. Finally, once guest authors realize they could be personally liable for the bodily injuries resulting from their misrepresentations, they and other potential guest authors will be deterred from engaging in such unethical and illegal behavior.

## False Claims Act

In addition to the claims for personal injuries caused by the guest authors' fraud, should the article constitute illegal off-label promotion by the pharmaceutical company, then the guest author may be held liable potentially as a conspirator under the federal False Claims Act (FCA) [32]. The FCA has effectively been used by private persons and the federal government to prohibit off-label promotion when company representations encouraged health care professionals to submit false payment claims to government health care programs. As recently reported by Kesselheim et al. [33], private individual actions under the FCA (also known as *qui tam* actions) allow company insiders and others with special knowledge of potential violations to initiate legal actions, which the government may join or take over. In particular, the FCA *qui tam* provision permits a private person, known as a relator, to file a lawsuit on behalf of the US government, on grounds that he or she has information that the named defendant has intentionally submitted, or instigated the submission of, false or fraudulent claims to the United States [34]. The relator, who need not have been personally affected by the defendant's demeanor, stands to receive a portion (usually about 15%–25%) of any recovered damages. A *qui tam* suit initially remains under seal for at least 60 days, during which the Department of Justice can investigate and decide whether to join the action.

This bounty system has acted as a powerful enticement in a variety of health settings, leading to a wellspring of FCA litigation [35], and such claims have become a usual feature of trials for off-label promotion [36],[37].

The Food, Drug, and Cosmetic Act (FDCA) governs drug safety; under it, manufacturers are forbidden from directly marketing a drug for a use other than the FDA-approved indication [38]. Under the FCA, lawsuits have been brought for FDCA violations against drug companies, based in part upon the company's utilization of ghostwritten articles to support illegal off-label use that induces physicians to prescribe medication for unapproved uses. In 2004, Pfizer pleaded guilty to charges that its Warner-Lambert unit flouted federal laws (FDCA and FCA) by promoting non-approved uses for a drug, alleging it used an illegal marketing strategy to drive up sales. Pfizer paid US$430 million in settlement, including US$24.6 million to the whistleblower who first reported the marketing manipulations. The lawsuit alleged that the Neurontin (gabapentin) marketing campaign included compensating doctors for putting their names on ghostwritten articles, paying them hefty speakers' fees, and covering the costs of “educational” trips at lavish resorts [39],[40].

Obtaining formulary coverage for off-label drug uses in the US can be especially hard, but approval can be advanced by articles supporting off-label use. If ghostwritten articles published by Medicare- and Medicaid-recognized peer-reviewed medical journals are used as clinical evidence to establish medically accepted indications for off-label drugs, they are arguably inducing prescriptions to be written and paid for by the US government under false pretenses. The ghostwritten articles may then form the basis for FCA claims [41],[42].

FCA inflicts civil liability against persons or entities presenting false payment claims or using false records or statements to get claims paid or approved or causing third parties to do so. Statutory damages include up to US$11,000 per false claim submitted (i.e., per each reimbursement submitted for an off-label indication), plus 3-fold damages for the government [43]. If the ghostwritten article causes physicians to prescribe a drug for off-label use to patients on government assistance, the prices paid by the government for these off-label prescriptions can be obtained as damages (and trebled) in a successful FCA prosecution. The potential that participating in a ghost authored article can result in liability for conspiracy under the FCA may be another deterrent to the unethical practice of guest authorship.

## Liability under the Anti-Kickback Statute

If it is established that, in consideration of prescribing the manufacturer's drug, the manufacturer agreed to the naming of the physician as a guest author, such arrangements would violate the federal Anti-Kickback Statute [44], the primary federal law governing physician-manufacturer consulting provisions. Enacted to protect Medicare and Medicaid programs against inappropriate use of services and unnecessary expenditures, it criminalizes suppliers inducing the use of products or services by providing remuneration to ordering physicians who knowingly offer, pay, solicit, or receive remuneration (in cash or kind, directly or indirectly, overtly or covertly) to induce (or in exchange for) the prescribing, purchasing, or recommending of goods or services reimbursable by any federal health care program [45].

Since paying physicians to become honorary or guest authors of a ghostwritten paper may influence their clinical judgment, subsequently increasing product sales (and government health care costs), and putting patients at risk by misrepresenting risk-benefit, both physicians and sponsor companies may be legally liable. The FCA, in conjunction with the Anti-Kickback Statute, can also be utilized to curb unethical ghostwriting. Via the FCA, a claim can be filed on behalf of the government by anyone possessing information regarding the anti-kickback violation and, if successful, the claimant or “relator” can share in any damages collected on behalf of the government. In addition, once the government is apprised of a kickback violation, the Department of Justice may bring criminal actions against violators of the Anti-Kickback Statute. Classified as a felony, the maximum individual punishments are fines of up to US$25,000 and imprisonment for up to five years [46]. Furthermore, individuals guilty of violating the statute can be excluded from participation in government programs such as Medicare and Medicaid [47]. The threat of civil and potential criminal prosecution is another potential manner of curbing guest authorship, especially when it is the result of reciprocal agreements between physicians/guest-authors to prescribe the drug and manufacturers promising to use the physician as a guest author.

## No Recourse to the First Amendment

In their defense, guest authors and pharmaceutical defendants may try to argue they have a First Amendment right to participate in ghostwriting. The US has a rich history of protecting anonymous speech, especially in the area of political speech [48]. However, the same level of protection does not apply to commercial speech, i.e., speech promoting the safety/sale of a drug. Moreover, the US Supreme Court has firmly held that “the First Amendment does not shield fraud” [49] and courts have consistently rejected such First Amendment arguments in cases in which drug companies have been sued for fraudulent or off-label promotion [50]. Accordingly, the First Amendment should not provide any sanctuary to guest authors and pharmaceutical companies engaged in fraudulent commercial speech.

## Conclusion

In addition to openly infringing academic standards and contributing to fraud, the crisis of credibility resulting from medical ghostwriting persists in published reports on the efficacy and safety of proposed treatments. The situation is so dire that the public is forced to seek judicial intervention to curb dangerous, unethical medical ghostwriting. Stakeholders, including universities, journals, pharmaceutical companies, and academic KOLs, have largely failed to heed public calls for honesty in reporting clinical research. Since these complaints have fallen on deaf ears, we believe the courts now have the task of restoring the integrity of the medical literature.

## Abbreviations

FCA: False Claims Act
KOL: key opinion leader
